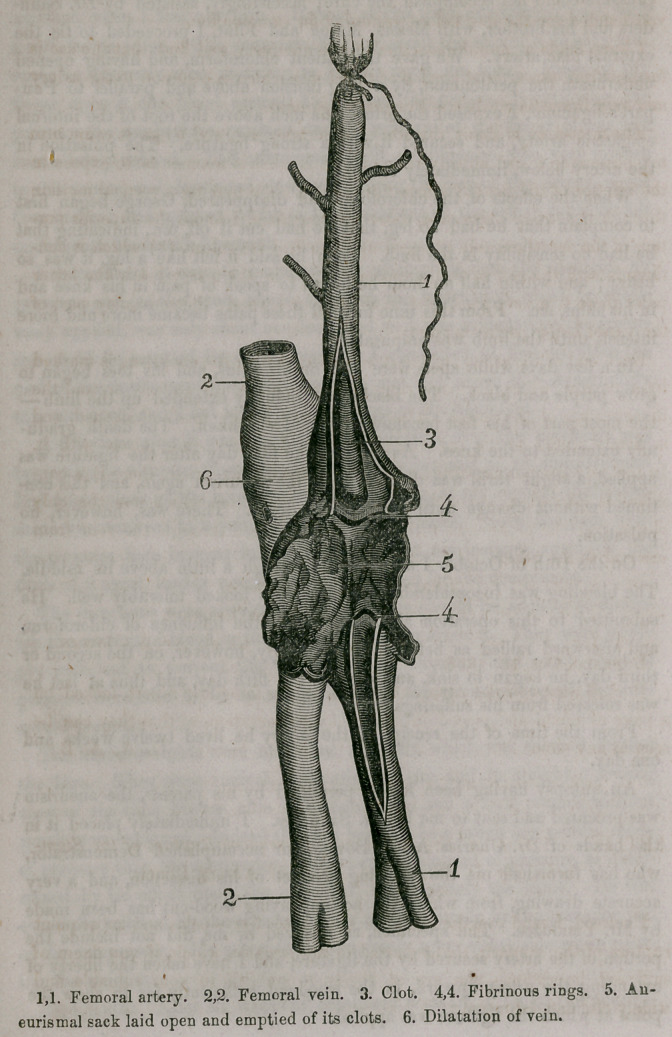# Traumatic, Femoral Aneurism

**Published:** 1857-01

**Authors:** Frank H. Hamilton

**Affiliations:** Buffalo


ART. III.— *Traumatic, Femoral Aneurism. Ligature of the External Iliac artery. Amputation. Fatal result.* By FRANK H. HAMILTON, M. D., Buffalo.

George Hoyt, an intelligent and interesting lad, æt. 14, residing at Houghton, in Allegany Co., N. Y., stabbed himself in his left thigh, accidentally, on the 28th of July, 1856, with the large blade of a pocket knife. The knife penetrated to the depth of about one inch and a half, at a point one inch below Poupart's ligament, and over the course of the femoral artery. The hæmorrhage was at the moment very profuse, but it ceased entirely within two minutes, yet not until he had fainted completely. It was estimated by several persons, that he lost in this short space of time, two quarts of blood. But I will permit one who was present during all his subsequent illness, to describe the principal events: "When the accident occurred the family were in the door-way and heard him exclaim, 'Ma, I am bleeding to death.' He came about two rods to meet them — they all ran to him — he spoke and said, 'I cannot see,' and immediately fainted. His eldest sister caught him in her arms, while the second hastened to loosen his clothes to find the place from whence the blood came. 'The blood,' she says, 'gushed out as from a spout.' She instantly closed his pants and pressed the hem snugly upon the spot. The eldest sister says the blood came in gushes, terrible gushes, through the unbuttoned front of the pantaloons. It could not have been more than two minutes before the blood ceased to flow, and almost as suddenly as it had commenced. In about thirty minutes George was able to speak in very faint tones. A messenger having been already despatched for Dr. Saunders, a very intelligent surgeon, residing eight miles distant, he was placed in a horizontal position upon a board, and carried into the house and laid upon a bed. All this time pressure was maintained steadily upon the wound, except when passing through the door; at which moment the fingers were removed, but the blood did not flow. This made us think that the pressure had nothing to do with stopping the blood, but that it was due to the fainting and to the cold water. When the doctor arrived he was better."

The remainder of the history of the case, up to the time of my first visit, I received from others who were present.

Dr. Saunders entertained some hope, from the conflicting accounts then given, of the appearance of the blood, &c., that only the femoral vein had been wounded; and as there was now no bleeding or pulsation, such as would indicate an aneurism, he applied a very simple dressing, securing it with a roller I ought to mention, also, that when I first saw the patient the wound seemed to be rather over the femoral vein than over the artery; or if it was over the artery it appeared to be upon its inner margin.

On the second or third, perhaps as early as sometime during the first day, an unusual pulsation was noticed by some members of the family. It then felt as if "the blood was close to the finger; with every beat it raised one half inch from the level of the skin; it seemed as if the blood was separated from the finger by the thinnest gossamer. If we pressed upon it a little time the tumor would for a while remain depressed. The beating, George said, was the worst when it first commenced, and each day it seemed to become less."

This occurred on Monday, July 28. Tuesday a chair was placed by the side of his bed, and he was helped into it while his bed was being made up. The same was done, probably, on Wednesday. Thursday, with the aid of crutches, he walked into the adjoining room. Friday, Saturday and Sunday, he walked about these two rooms. Monday he walked to the horse barn. Tuesday and Wednesday he was more quiet.

I am not informed that he was seen by any physician during all this time, except as mentioned on the first day. The distance at which Dr. Saunders lived, and the absence of any lively apprehension of danger, led to this neglect; yet it is not supposed that any more active interference, even at this early period, could have prevented the final result. It is remarkable, however, considering that the artery had been completely severed, and considering that the wound of the skin, half an inch in breadth, had so recently closed—it is certainly remarkable that with all these circumstances existing, and with his daily exercise, it did not give way and in a moment terminate his life. But this is scarcely less remarkable than that it should ever have ceased to bleed spontaneously, or with such apparently insufficient means as were at first employed by the terrified assistants.

On Wednesday, Aug. 6, ten days after the accident, a compress was, for the first time, applied. On Thursday, a messenger having been sent to me for advice, Charriere's compressor was substituted, but proving very painful, the simple compress, with a bandage, was, after a few hours, again employed; and this was continued until Saturday, Aug. 9th, the thirteenth day of the accident, when I first saw him. He was, at this time, exceedingly pale, with a soft, feeble pulse. The aneurism, then, of about the size of a hickory-nut, could be distinctly seen pulsating as he lay upon his back. Pressing my finger upon it, the blood seemed to be near the surface. Continuing the pressure the sac became emptied, and on removal of the finger it was not immediately refilled. The aneurismal thrill or vibration, was very intense in the tumor, and continued downward along the line of the artery, about four inches; and upward, as far as the pulsations of the artery could be felt underneath Poupart's ligament.

I determined at once to make another attempt to close the aneurism by pressure, and as the space above, to which the instrument could be effectually applied, was only about one inch, and as it was evident that the aneurism could be emptied by direct pressure, I concluded at first to place the pad of the instrument over the aneurism and not above it. Accordingly this was done, and I left him again in charge of Dr. Saunders.

A few days after it was found that the cicatrix, over the aneurism, was beginning to ulcerate, from the constant pressure; and as no improvement had taken place in the condition of the aneurism, at my request the instrument was removed to a point directly over the pubis. Soon the pain from the pressure here became insupportable, and at my instance again, on the 30th of August, leaden weights were substituted for the compressor.

The directions were always that the pressure should be such as to diminish the current of blood in the artery, but not to arrest it. They were to apply sufficient to prevent the "thrill." The direction, and exact point of pressure were also to be as much varied as the circumstances of the case would permit.

The leaden weights were made fast in a belt, which was secured around the hips. They were conical, and varied in size and in weight. During most of the time he was able to sustain seven and a half pounds without much inconvenience. Indeed this was found to be a much less painful mode of pressure than the compressor, even when the amount of pressure, as determined by its effect upon the aneurism, was precisely the same. He was especially relieved by the removal of the pressure made by the posterior pad of the compressor. He was able to turn partly upon either side while wearing the weights.

Sept. 19th. After six weeks of continued pressure, I found the aneurismal tumor and the thrill as when I first saw it. The lad was becoming gradually more feeble and his left limb œdematous. It was apparent that pressure could not accomplish the cure; accordingly, assisted by Dr. Saunders and his brother, with Messrs. Mason and Flint, I proceeded to tie the external iliac artery. We gave the patient chloroform, and having opened underneath the peritoneum, by a long incision above and parallel to Poupart's ligament, I exposed the artery one inch above the root of the internal epigastric artery, and secured it with a strong ligature. The pulsation in the artery below, immediately ceased.

When the effects of the chloroform had disappeared, George began first to complain that he had no leg, that we had cut it off, &c., indicating that he had no sensibility in the limb. Soon he said it felt like a log, it was so heavy; and within half an hour he began to speak of pain in his knee and in his shins, &c. From this time forward these pains became more and more intense, until the limb was amputated.

In a few days white spots were seen on his shins, and his toes began to grow purple and black. The blackness gradually extended up the limb—the most part of his foot becoming dry and shrunken. The death gradually extended to the knee. As early as the fifth day after the ligature was applied, a slight thrill was discovered in the aneurism again, and this continued without change until the time of death. There was, however, no pulsation.

On the 16th of October I amputated his thigh a little above its middle. The bleeding was inconsiderable, and the flaps looked tolerably well. He submitted to this operation also, while under the influence of chloroform, and afterward rallied as before. Subsequently, however, on the second or third day, he began to sink, and died on the fifth day, and thus at last he was released from his sufferings.

From the time of the receipt of the injury he lived twelve weeks and one day.

An autopsy having been kindly permitted by his parents, the aneurism was procured and sent to me by Dr. Saunders. I immediately placed it in the hands of Dr. Charles Ap A. Bowen, our accomplished Demonstrator, who has furnished me the following account of his dissection, and a very accurate drawing, from which the accompanying wood-cut has been made by Mr. Vanduzee. The specimen, as received by me, did not include the portion of the artery secured by the ligature, and I have taken the liberty of extending the artery upward, in the wood-cut, sufficiently far to show the point at which the ligature was applied.

I have omitted to mention that the ligature came away on the 16th of Oct., the day on which I made the amputation.

1,1. Femoral artery. 2,2. Femoral vein. 3. Clot. 4,4. Fibrinous rings. 5. Aneurismal sack laid open and emptied of its clots. 6. Dilatation of vein.

F. H. Hamilton, M. D.,

*Dear Sir:* I send you a drawing and a description of the post-mortem appearances presented by the case of aneurism of the femoral artery, which you handed me the day before yesterday for examination.

I have made a careful dissection of the aneurism and find that the sac is formed from the arterial portion of the sheath which is common to the artery and vein immediately below Poupart's ligament.

The aneurism is about the size of a hickory nut, and when it came into my possession it had already been opened in such a manner as to expose in the interior of the sac (5) a firm fibrous clot. On making a vertical incision through the artery above and below the aneurism, I found the superior portion of the vessel entirely plugged up by a firm coagulum, (3) which is continuous with the clot in the aneurism—while in the artery below the latter point there is a very small clot in the upper portion: the remaining part of the vessel being perfectly empty.

Each end of the artery, where it is connected with the aneurism, is marked by a narrow, white, fibrinous ring (4,4) which extends entirely around the inner surface of the vessel. The femoral vein is very much thickened, and is firmly united to the aneurismal sac, but does not communicate with it. In the superior portion of the vein, just above the aneurism, there is a considerable dilatation, a pouch, containing a coagulum, which was the only obstruction the probe met with when it was passed through the vein from one end to the other.

I have, Sir, the honor to be,

Your obed't servant,

Chas. Ap A. Bowen.

Buffalo, Oct. 12, 1856.

P. S. I send you the parts referred to in the foregoing report, tacked on a piece of board and placed in alcohol for preservation, so that they can be seen at any time. C. A. A. B.

## Figures and Tables

**Figure f1:**